# Potential Risks of Plant Constituents in Dietary Supplements: Qualitative and Quantitative Analysis of *Peganum harmala* Seeds

**DOI:** 10.3390/molecules26051368

**Published:** 2021-03-04

**Authors:** Matilde Calderoni, Maddalena Altare, Luca Mastracci, Federica Grillo, Laura Cornara, Aldo Pagano

**Affiliations:** 1 Department of Experimental Medicine (DIMES), University of Genoa, 16132 Genoa, Italy; calderoni.matilde@gmail.com (M.C.); maddalena.altare@gmail.com (M.A.); 2 Pathology Unit, Department of Surgical and Diagnostic Sciences (DISC), University of Genoa, 16132 Genoa, Italy; luca.mastracci@unige.it (L.M.); federica.grillo@unige.it (F.G.); 3 IRCCS Ospedale Policlinico San Martino, 16132 Genoa, Italy; 4 Department of the Earth, Environment and Life Sciences (DISTAV), University of Genoa, 16132 Genoa, Italy

**Keywords:** dietary supplement, microscopy, real-time RT-PCR, sophistication

## Abstract

The free online trading of herbal mixtures useful for various purposes facilitates the circulation of dangerous herbs or plant parts. This is the case, for example, of the illegal trade in seeds of *Peganum harmala* (*Pgh*), which contain alkaloids capable of inhibiting monoamine oxidase (MAO) and are therefore used in hallucinogenic preparations, such as the psychedelic drink *ayahuasca*. The precise identification of these seeds and their distinction from other very similar but not dangerous seeds are necessary for forensic purposes and represents an advance in avoiding the adulteration of mixtures. In this work, we show the qualitative identification of *Pgh* seeds by optical and electron microscopy and the parallel development of a real-time qPCR test, which reveals, in a species-specific manner, the presence of *Pgh* DNA up to quantities lower than 1 pg. In addition to the species specificity and high sensitivity, the reaction accurately quantifies the presence of seeds or parts of seeds of *Pgh* in complex herbal mixtures, thus giving an indication of the danger or otherwise of the product.

## 1. Introduction

*Peganum harmala* L. (*Pgh*), belonging to the Zygophyllaceae family, is commonly known as *Syrian rue*, *Harmel*, *Esfand* or *Espænd* (in Persian), and Yüzerlik (in Turkey). This herb is native to the Middle East, Africa, and the Mediterranean area and used in traditional medicine to treat different diseases. *Pgh* seeds and fruits are mainly used as digestive, diuretic, hallucinogenic, hypnotic, antispasmodic, emetic, and abortifacient agents [1]. *Pgh* also shows analgesic and anti-inflammatory properties due to the presence of alkaloids that act on the nervous system, probably mediated by opioid receptors [2]. In addition, *Pgh* is used in rites to ward off the evil eye because of an old belief in Iran and Turkey [3,4].

The plant contains beta-carboline alkaloids (harmine, harmaline, and tetrahydroharmine), which stimulate the central nervous system by inhibiting the metabolism of amine neurotransmitters, in particular monoaminoxidases. This can cause toxic effects, namely neurosensorial symptoms, hallucinations, locomotor ataxia, nausea, vomiting, confusion, and agitation, as well as cardiovascular disorders such as bradycardia and low blood pressure [3].

Several cases of intoxication have been reported consequent to the ingestion of *Pgh* seeds used as a traditional medication, mainly for its sedative and emmenagogue properties [4,5,6,7,8].

Achour et al. reported information on 200 cases of intoxication collected at the Centre Antipoison et de Pharmacovigilance du Maroc during the period 1984–2008 [9]. In addition, a recent case of fatal poisoning involving a woman reported in Algeria was caused by the injudicious use of a preparation made of honey, oil, and crushed *Pgh* seeds [10].

In other cases, intoxications were due to the combined use of *Pgh* with other plant ingredients, such as in herbal infusions [11]. Brush et al. reported a case of severe intoxication caused by the use of *Pgh*, together with the hallucinogenic 5-methoxydimethyltryptamine (5-MeO-DMT). More recently, another case of poisoning due to intentional ingestion of a self-made infusion of *Phg* seeds bought over the internet has been described in Italy. Moreover, seeds are present in “legal highs” such as incense and smoking blends [12] and have become popular among young people as a substitute of *Banisteriopsis caapi*, due to the presence of similar harmala alkaloids [13], in the preparation of the psychedelic drink *anahuasca*, an analogue of the Amazonian *ayahuasca*.

In some European countries, such as Turkey, the use of *Pgh* seeds in herbal preparations and supplements is legal, while in others it is banned. *Pgh* seeds are not included in the list of plants eligible for use in food supplements, known as BELFRIT, drawn under the cooperation project between Belgium, France, and Italy [14]. Conversely, other seeds similar for morphological features and size to those of *P. harmala* are legally inserted in the BELFRIT list and are legally used as ingredients of supplements in many European countries. This is the case of *Nigella sativa* (*Ns*) seeds, commonly known as black seeds, used in the treatment of different diseases throughout the world and very popular in traditional medicines such as Unani and Ayurveda [15].

Hence, there is a risk that *Pgh* seeds be misidentified with those of other plants, especially when they are part of complex herbal blends. Alongside this, it is of capital importance that methods to identify and to quantitatively determine *Pgh* seeds in complex vegetable mixtures are developed. In particular, the dietary supplement Berkap capsules, sold in Turkey and indicated to cure hemorrhoids, contain a mixture of black myrobalan, black sesame, senna, harmel (*Pgh*), elder flower, shepherd’s purse, pollen, horse chestnut, propolis, yarrow herb, and *Lathyrus*.

This study aimed at developing a protocol of identification of *Pgh* seeds in complex herbal mixtures. Our work involved several steps: first, characterization and comparison of the morphology and anatomy of *Pgh* and *Ns* seeds, by light and scanning electron microscopy (SEM); second, identification and quantification of the relative amount of the different seeds in the mixture, by using real-time qPCR molecular techniques.

At present, available methods to discriminate between the two species are based on nucleic acid sequencing (DNA barcoding) or on pharmacognostic techniques, such as phytochemistry and macro- and micromorphological characterization. Unfortunately, both DNA barcoding and microscopy techniques are mostly qualitative, and they do not allow the accurate determination of the percentage of *Pgh* seeds in a complex mixture. Moreover, chemical methods were developed to quantify harmala alkaloids in plant tissue extract, such as high-performance liquid chromatography (HPLC) or liquid chromatography (LC)–mass spectrometry, but the sampling procedure can last too long, or the experimental reproducibility can be too poor. Monsef-Esfahani et al. [16] developed a high-sensitivity HPLC method able to measure harmala alkaloids, and Wang et al. [17] combined HPLC with ion mobility spectrometry to obtain higher resolution for the analysis of alkaloids; despite this, the quantification was obtained by seeds of *Pgh* and not complex plant extracts, making the analysis less interesting for forensic applications.

Measuring the relative amount and concentration of a particular plant or plant part (botanical) within a herbal supplement can be useful for product standardization and to avoid health risks. The real-time qPCR adopted here has high qualitative and quantitative specificity and is able to detect the presence of the seeds of a particular plant species in complex mixtures containing morphologically similar seeds. Our interdisciplinary study can be useful for many fields of application, such as quality control and standardization of food supplements, as well as forensic investigations.

## 2. Results

### 2.1. Macro- and Micromorphology and Anatomical Features Allow to Qualitatively Distinguish between Pgh and Ns Seeds

The morphological features of *Pgh* seeds were initially considered. Due to similar shape and size, these seeds can be easily confused and exchanged with those of other plant species permitted in herbal supplements in most European countries and widely used in the kitchen to flavor foods. Examples include the seeds of *Linum usitatissimum* (flax or linseed), *Nigella sativa* (black cumin), *Carum carvi* (caraway), and *Sesamum indicum* (sesame).

**Figure 1:**
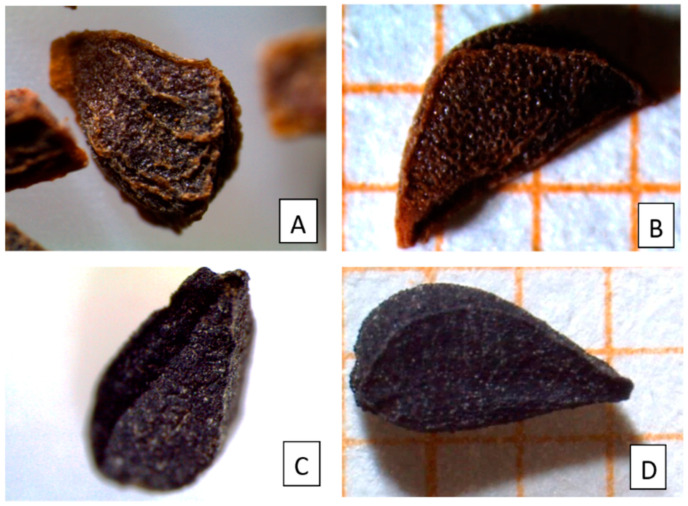
Light microscopy (LM). Comparison between color, shape, size, and seed coat morphology of *Peganum harmala* L. (*Pgh*) seeds (**A**,**B**) and *Nigella sativa* (*Ns*) seeds (**C**,**D**). Both seeds are subtriangular and show a seed coat with a rough surface and a reticulate ornamentation, more evident in *Pgh*. (**B**). Seed coat appearing brown to blackish-red in *Pgh* (**A**,**B**) and black in *Ns* (**C**,**D**).

**Figure 2:**
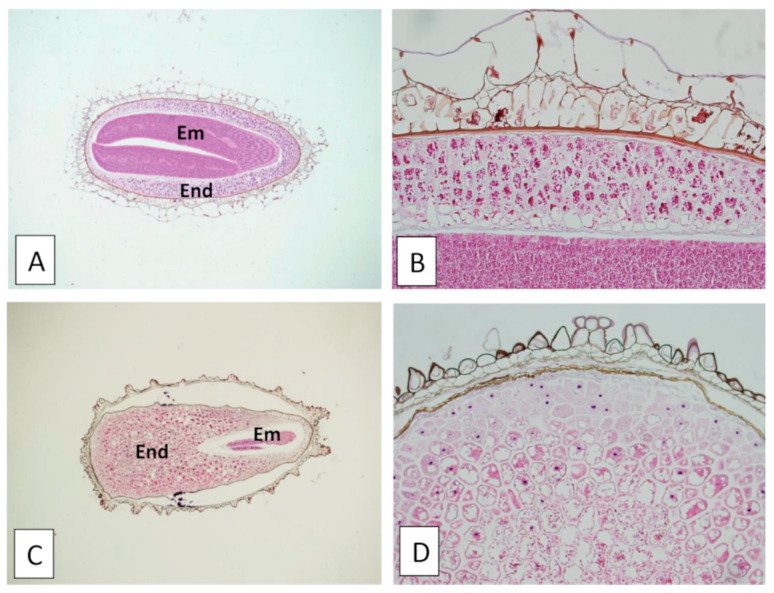
LM micrographs of histological sections of *Pgh* seeds (**A**,**B**) and *Ns* seeds (**C**,**D**) stained with hematoxylin–eosin. General features of seed coat structure, endosperm, and embryo are shown at 4× (**A**,**C**). Differences in seed coat morphology are highlighted at higher magnification (20×) (**B**,**C**). Em—embryo; End—endosperm.

In particular, the morphology and the anatomy of *Pgh* seeds were compared with those of *Ns* (*N. sativa*) seeds (Figure 1 and Figure 2), widely used in many dietary supplements [18] and commonly employed as a spice [19]. *Ns* seeds have a long history of medicinal use, so much so that they are also mentioned in the Bible and in the words of the Prophet Mohammed [20].

In Figure 1A–D, seeds of *Pgh* (A and B) and *Ns* (C and D) are compared, showing similar subtriangular shape and similar size. *Pgh* seeds are tetrahedral-triangular, have a laterally curved, peaked edge, are brown to blackish-red in color, and have a size of 2.0–3.5 × 1.5–3.0 mm. The seed surface is covered with polygonal reticulations, while three or four ridges surround the entire seed length (Figure 1A,B). *Ns* seeds have a trigonous-ovate shape, blackish rough surface, three longitudinal ridges, pointed ends, and their size is 2.0–3.0 × 1.5–2.5 mm (Figure 1C,D).

In addition, microscopic analysis of the powdered commercial dietary supplement Berkap revealed several fragments of *Pgh* seeds, well recognizable by the typical reticulation and brown-reddish color of the seed coat.

Comparison of hematoxylin–eosin-stained histological sections revealed differences in seed coat structure and embryo/endosperm volumetric measures between *Pgh* seeds (Figure 2A,B) and *Ns* seeds (Figure 2C,D). The seeds’ longitudinal sections (Figure 2A,C) showed the presence of a larger embryo in *Pgh* than in *Ns*, while, on the contrary, a more developed endosperm (seed reserve tissue) was found in *Ns* compared with *Pgh*.

**Figure 3:**
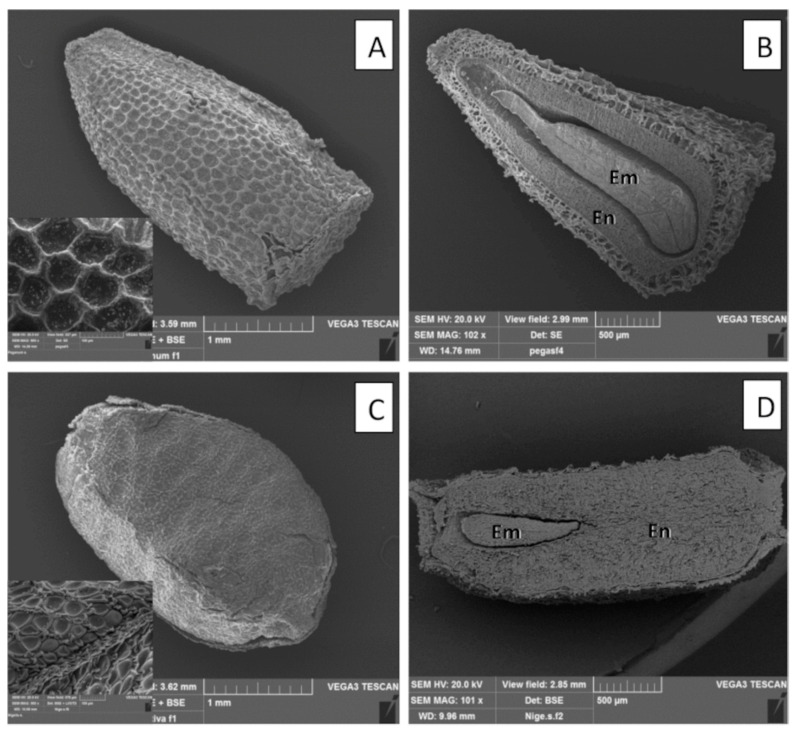
SEM micrographs showing general features (**A**), seed coat magnification (**A**, insert), and longitudinal section (**B**) of *Pgh* seeds, and general features (**C**), seed coat magnification (**C**, insert), and longitudinal section (**D**) of *Ns* seeds. Em—embryo; End—endosperm.

SEM analyses showed the typical sculpture of the seed coat, with a well-visible reticulate pattern in *Pgh* seeds (Figure 3A and insert), corresponding to the tubular to concave type, characterized by polygonal cells and reticulate tectum, according to the classification of Bartholtt and Ehler [21] and the observations of Ib and Yankova-Tsvetkova [22]. The seed coat of *Ns* (Figure 3C and insert) showed the typical collapsed (ocellate) prismatic cells, while ligulate columellate cells form small and short ridges running across the surface, according to what Heiss et al. [23] reported. In addition, SEM analyses confirmed the differences in the embryo/endosperm volumetric relationship between *Pgh* and *Ns* seeds (Figure 3B,D).

Qualitative analysis by macro- and microscopic investigations revealed the presence of entire *Pgh* seeds or their remnants within the dietary herbal mixture. However, this method could not quantify their abundance into the sample. Therefore, a highly sensitive molecular method was used for this purpose.

### 2.2. A DNA Region Internal to the Plastid Maturase K Gene Allows to Recognize Species-Specifically Pgh by qPCR

Our aim was the development of a method that allows to unequivocally identify *Pgh* in a herbal mixture (also in traces) and simultaneously determine its relative amount with respect to other seeds present in the same blend. Indeed, such an unexpansive and fast method is dictated by the need to determine the relative dangerousness of different mixtures. We therefore decided to develop a very sensitive qPCR system with a fluorescent probe.

With these assumptions, we decided to amplify a region of plastid DNA. Plastid DNA sequences are generally used for a DNA barcoding technique in a non-quantitative manner, in particular ribulose bisphosphate carboxylase large chain (*rbcl)* and maturase K *(matK)* loci [24]. Plastid DNA is present in high-copy numbers in every cell, making the qPCR reaction highly sensitive to finding even traces of the seeds. For this purpose, after sequence analysis we decided to use an internal region of the *matK* gene, known to have a high species resolution (Figure S1).

**Figure 4:**
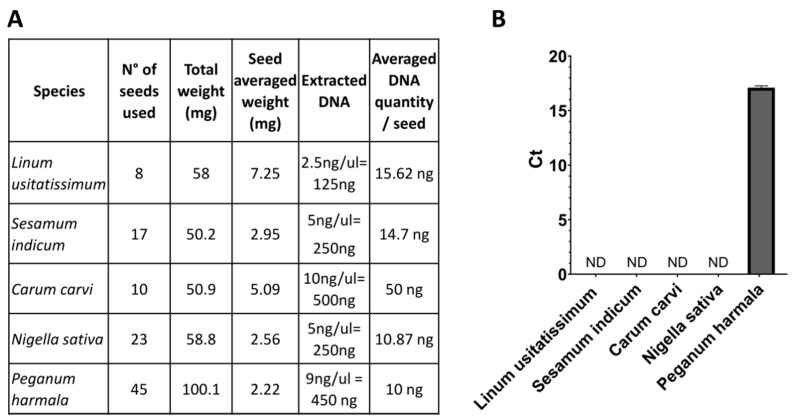
Species-specificity validation of *Pgh* detection. (**A**) Description of samples used for the qPCR reaction. (**B**) Real-time RT-PCR quantitative determination of *matK* specific for *Pgh*.

To guarantee the specificity of this reaction only in the presence of *Pgh*, we carried out the same qPCR reactions also on seeds of *Linum usitatissimum*, *Sesamum indicum*, *Nigella sativa*, and *Carum carvi*, which have similar seed morphology and can be easily confused with *Pgh*. The results shown in Figure 4 indicate that the *Pgh* probe does not amplify any of the control species even at high sample quantities. Therefore, this experiment demonstrates the species specificity of the amplicon chosen for the qPCR reaction to detect only *Pgh* DNA.

### 2.3. Detection and Quantification of Pgh Seed Traces in Complex Herbal Mixtures

**Figure 5:**
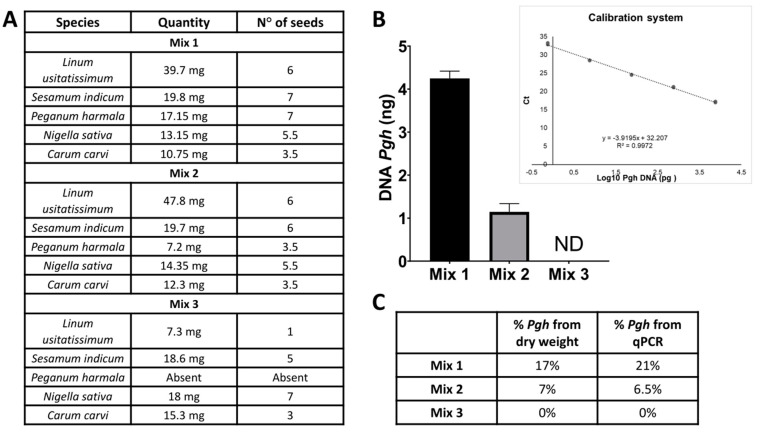
(**A**) Summary table of weight and averaged number of seeds used for each species in three mixtures. (**B**) Real-time RT-PCR quantitative determination of *Pgh* in three different mixtures. (**C**) Comparison between the percentage of *Pgh* seed quantity versus the other seeds and the percentage of *Pgh* DNA (compared to total DNA extracted) found with PCR analysis.

To assess if the reaction discriminates quantitatively very faint traces of *Pgh* in complex mixtures (thus resembling possible supplements or blends for hallucinogenic use available in the network), we generated three different combinations of various seeds, from which DNA was extracted (Figure 5A). From mixture 1, we obtained 19.5 ng/µL of DNA, whereas from mixtures 2 and 3, we obtained 17.5 and 16 ng/µL, respectively.

We then built a calibration line that would match amplification values (cycle thresholds (Ct)) with quantities of DNA measured by spectrophotometry. Moreover, we considered that DNA extractions from different mixtures could lead to different yields. Therefore, we focused on the evaluation of the percentage of *Pgh* DNA with respect to the total mixture, and then we compared it to the dry weight percentage of *Pgh* in the blends.

The results obtained indicate that the reaction highlights 0.75 pg of *Pgh* DNA in 100 mg of the extracted sample, thus demonstrating a remarkable species-specific sensitivity of the assay and the accurate determination of the percentage of *Pgh* seeds in a mixture (Figure 5B,C).

Therefore, we consider this method suitable to evaluate the possible dangerousness of a herbal mixture or a dietary supplement.

### 2.4. Proof of Concept/Field Testing: Detection and Quantification of Pgh in Commercial Products

**Figure 6:**
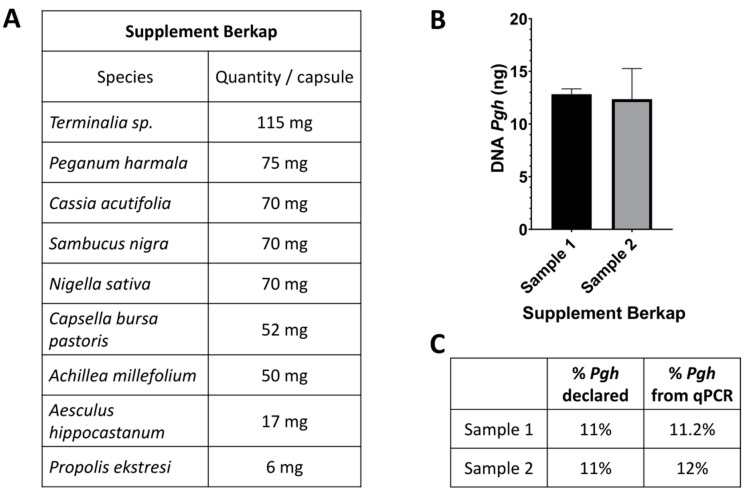
(**A**) Summary table of Berkap ingredients. (**B**) Real-time RT-PCR quantitative determination of *Pgh* in two samples of the supplement Berkap. (**C**) Comparison between the percentage of *Pgh* quantity declared and the percentage of *Pgh* DNA (with respect to total DNA extracted) detected.

To evaluate the practicality and reliability of the assay in the analysis of a commercial herbal blend that can be purchased on the internet, we analyzed a dietary supplement in capsules (Berkap BSR Kapsul) (Figure 1E). The commercial preparation indicates the relative quantities of each of the nine components present in each capsule (Figure 6A). After extracting the DNA from two independent capsules, we measured the amount of *Pgh* and its relative percentage with respect to the other species; next, we compared the results with what was declared on the package. The results showed that the assay detects *Pgh* seeds in a complex mixture in a quantitative manner (Figure 6B). Indeed, the relative quantity of *Pgh* declared by the supplier (11%) was detected by the test with an accuracy greater than 90%, thus validating the suitability of this system to analyze commercial herbal blends (Figure 6C).

## 3. Discussion

With the development of the dark web, the widespread distribution of prohibited or dangerous substances of plant origin such as “smart drugs” has progressively increased [25]. At the same time, herbal products used as herbal remedies and food supplements are becoming more and more popular worldwide due to increasing interest in natural therapies [26]. Herbal products are a mixture of two or more herbs [27]. Consequently, it is evident that the identification of potentially toxic or illegal plant species within these products is necessary to ensure public health and promote safety. Many of these herbal formulations, available in the market without prescription and manufacturer’s claims, are described as “natural,” but may contain dangerous or illegal synthetic substances or potentially toxic plant species [25,28]. Another problem is represented by the intentional adulteration of herbal products, involving partial or total replacement of the original herb with other herbal materials of lower therapeutic value. This adulteration is frequent when the morphological features of the original herb are similar to those of less expensive herbal drugs [28]. In addition, indirect adulteration can also occur due to deficient plant knowledge of manufacturers [29].

Therefore, reliable and rapid methods for a correct identification and quantitative evaluation of herbal drugs present within dietary supplements and herbal remedies is needed to ensure product safety and standardization.

The aim of our study was to combine microscopic and molecular techniques to obtain a rapid, qualitative, and quantitative analysis of complex herbal mixtures. 

For this purpose, *Peganum harmala* seeds were compared with other morphologically similar seeds, in particular those of *Nigella sativa. Pgh* use is considered illegal for food preparation in some European countries because it contains psychoactive substances: actually, four grams of *Pgh*, equal to about 2000 seeds, is as potent as 200 mg of harmine, a hallucinogenic alkaloid that can block the action of serotonin. Moreover, in combination with plants containing dimethyltryptamine (DMT) or other tryptamine hallucinogens, it can produce hallucinogenic effects. However, the intensity of the effects varies widely with the individual, thus making impossible the creation of a real guideline for a threshold value that results in intoxication or death.

Microscopy is a traditional method reported in the pharmacopoeia for plant identification. In a recent review, Ichim et al. [30] examined the authenticity of 508 microscopically authenticated herbal products, sold in different countries, concluding that microscopy is a rapid and cost-efficient method and can cope with mixtures and impurities. We used both light and electron microscopy to visualize the peculiarities of *Pgh* seeds, in order to provide information to use in customs laboratories (for example), where microscopes could be of one type or another. Obviously, SEM analysis can offer more appreciable details than light microscopy, but the cost of this technology can be an issue for some laboratories. However, combinations with chemical or DNA-based techniques are necessary to obtain a quantification of the investigated substance.

Extremely sensitive methods to identify traces of plant species among others are required to prevent adulteration. Moreover, DNA barcoding has been used for this purpose; this technique enables to discriminate between the presence or the absence of the investigated DNA in a mixture, without, however, quantifying the amount under consideration and the consequent dangerousness.

The qPCR system is simple and cheap, and the necessary instrument is commonly present in almost all laboratories (customs, analysis laboratories, etc.). This technique allows several replicas to be carried out, thus bringing experimental results to greater reliability. Most importantly, in contrast to the DNA barcoding approach, qPCR helps to quantify the sample presence, allowing to detect if a substance exceeds the maximum level allowed by law, for example.

In this work, we developed a species-specific reaction, highly sensitive and able to identify the presence of *Pgh* up to less than 1 pg. We used an internal region of *matK*, a chloroplast gene generally chosen for its high species resolution.

Thanks to its specificity and high sensitivity, this reaction is therefore suitable for use in the forensic field to identify traces of this MAO inhibitor, which is illegal in some countries but hidden in complex mixtures for recreational use. 

Furthermore, the quantitative precision and the possibility of determining the relative quantities of *Pgh* in blends makes this reaction useful for the determination of the real dangerousness (and the effective MAO-inhibiting power) in psychoactive mixtures rather widespread.

## 4. Materials and Methods

### 4.1. Plant Material

Seeds of *Pgh* from Moroccan local markets were kindly provided by Giorgio Samorini, an independent ethnobotanist expert in the traditional use of intoxicating plants. Seeds of *Linum usitatissimum* (flax or linseed), *Nigella sativa* (black cumin), *Carum carvi* (caraway), and *Sesamum indicum* (sesame) were obtained from commercial products, since they are widely used as a flavor in bread, cakes, salads, cheese, and sauerkrauts.

The herbal mixture contained within the capsules of the dietary supplement Berkap (Aksuvital, Shiffa Home, Istanbul, Turkey) was also used to test the capability of microscopic and molecular techniques to identify and quantify *Pgh* seeds into a complex matrix.

### 4.2. Macro- and Micromorphological Analyses

Macro- and micromorphological features of *Pgh* and *Ns* seeds were analyzed and compared by light (LM) and scanning electron microscopy (SEM).

#### 4.2.1. Light Microscopy (LM)

Seeds of *Pgh* and *Ns* were observed using a stereomicroscope to compare size, color, and seed coat morphology (Figure 1A,B). In addition, the powdered mixture contained in Berkap capsules was also examined, identifying seed fragments of *Pgh* on the basis of seed coat morphology (Figure 1D).

For micromorphology, the seeds under investigation were hydrated for 24 h by dipping in spring water in a 37 °C controlled temperature stove. After hydration, the seeds were fixed for 24 h in 10% buffered formalin. For each seed type, five plastic embedding blocks, each one containing three seeds, were produced. The samples were then processed by an automated processor, Leica ASP 6025 (Leica Biosystems, Wetzlar, Germany), which in a single work cycle of approximately 8 h permits alcohol dehydration, xylene substitute clarification and paraffin embedding. Paraffin blocks were produced using the Leica DP 500 embedding station. For each paraffin block, three 4 micron thick sections were microtome-cut (Leica RM 2255 microtome, Leica Biosystems, Heidelberg, Germany) and stained in hematoxylin and eosin using an automated stainer (HE600, Ventana Medical Systems, Oro Valley, AZ, USA). Microphotographs were taken using the Olympus DP70 (Olympus Life Science, Tokyo, Japan) camera mounted on an Olympus BX51 optical microscope.

#### 4.2.2. Scanning Electron Microscopy (SEM)

Seeds of *Pgh* and *Ns* were fixed overnight at 4 °C in FineFIX working ethanolic solution (Milestone S.R.L., Bergamo, Italy), as suggested by Chieco et al. [31]. The specimens were then dehydrated with a graded series of ethanol (80%, 90%, 95%, and 100%) and exposed to drying until the critical point of CO_2_ was reached by using a critical point drier apparatus (K850 CPD 2M Strumenti S.R.L., Roma, Italy). The dried samples, mounted on stubs and coated with 10 nm gold, were observed with a Vega3 Tescan LMU scanning electron microscope (SEM) (Tescan USA Inc., Cranberry Twp, PA, USA) at an accelerating voltage of 20 kV.

### 4.3. DNA Extraction

The seeds were collected in a sterile mortar and ground with liquid nitrogen. Genomic DNA was isolated from 50–100 mg of seeds using DNeasy Plant Pro Kit (Qiagen, Hilden, Germany), according to the manufacturer’s instructions. DNA was then measured by Biophotometer Plus (Eppendorf, Hamburg, Germany) and stored at −20 °C.

### 4.4. Real-Time Quantitative RT-PCR Analysis

The total gDNA from samples was measured by real-time quantitative RT-PCR using the PE ABI PRISM^®^ 7700 Sequence Detection System (Perkin Elmer Corp./Applied Biosystems, Foster City, CA, USA).

The primer/probe set targeting the *maturase K* gene was designed on a sequence specific for *Peganum harmala*. The sequence of the gene was provided by GenBank (www.ncbi.nlm.nih.gov/Genbank, accessed on 29 January 2021; Accession Number AY177667.1). Sequences unique to *Pgh* were compared with those of closely related species.

The primers and a TaqMan fluorogenic probe were designed using Primer Express Software 3.0 (Applied Biosystems, Foster City, CA, USA) and validated using NCBI BLAST (Basic Local Alignment Search Tool).

The sequences for the *maturase K* gene were as follows: forward primer 5′-TGTGGTCGCAACCAGGAA-3′; reverse primer 5′-CCCAAGGATTTAGTTGCACATTG-3′; probe 5′-FAM-TCCATATAAATCACCTATCCAAACA-3′-TAMRA. 

The 2x TaqMan Universal PCR Master Mix (Applied Biosystems) was used for this analysis. The optimal primers and probe concentrations amounted to 0.6 µM and 0.2 µM, respectively.

Conditions for amplification were as follows: 1 cycle of 50 °C for 2 min, 1 cycle of 95 °C for 10 min, followed by 45 cycles of 95 °C for 15 s, 50 °C for 30 s, and 60 °C for 35 s.

The threshold cycle (Ct), which correlates inversely with the target DNA levels, was measured during PCR amplification as the cycle number at which the reported fluorescent emission increases above a threshold level. Genomic DNA levels were determined from the relative standard curve built from stock DNA dilutions.

## Data Availability

The data presented in this study are available on request from the corresponding author.

## Supplementary Materials

The following are available online. Figure S1: Identification of species-specific *matK* region. Three candidates amplicons for *Pgh matK* detection. The forward and reverse primers are colored in blue, while the FAM-TAMRA probe is marked in red. The chosen amplicon is described in Figure S1C.

## Sample Availability

Samples of the seeds are available from the authors.

## References

[1] Dube A., Misra P., Khaliq T., Tiwari S., Kumar N., Narender T. (2011). Therapeutic Potential of Harmala (Peganum harmala L.) Seeds with an Array of Pharmacological Activities. Nuts and Seeds in Health and Disease Prevention.

[2] Farouk L., Laroubi A., Aboufatima R., Benharref A., Chait A. (2007). Evaluation of the analgesic effect of alkaloid extract of Peganum harmala L.: Possible mechanisms involved. J. Ethnopharmacol..

[3] Frison G., Favretto D., Zancanaro F., Fazzin G., Ferrara S.D. (2008). A case of β-carboline alkaloid intoxication following ingestion of Peganum harmala seed extract. Forensic Sci. Int..

[4] Moshiri M., Etemad L., Javidi S., Alizadeh A. (2013). Peganum harmala intoxication, a case report. Avicenna J. Phytomed..

[5] Ben Salah N., Amamou M., Jerbi Z., Ben Salah F., Yacoub M. (1986). Un cas de surdosage en *Peganum harmala* L.. J. Toxicol. Clin. Exp..

[6] Mahmoudian M., Jalilpour H., Salehian P. (2002). Toxicity of Peganum harmala: Review and a Case Report. Iran. J. Pharmacol. Ther..

[7] Sadr Mohammadi R., Bidaki R., Mirdrikvand F., Mostafavi Yazdi S.N., Yazdian Anari P. (2016). *Peganum harmala* (Aspand) Intoxication: A Case Report. Emerg. Tehran.

[8] Ataee Z., Dadpour B., Rahimpour M., Najari D., Najari F. (2018). Acute Poisoning With Peganum Harmala, Esfand: A Rare Case Report. Int. J. Med. Toxicol. Forensic Med..

[9] Achour S., Rhalem N., Khattabi A., Lofti H., Mokhtari A., Oulaymani A., Turcant A., Bencheikh R.S. (2012). L’intoxication au Peganum harmala L. au Maroc: À propos de 200 cas. Therapie.

[10] Djafer R., Akil Dahdouh S., Boukachabia R., Megueddem M. (2017). À propos d’un cas d’intoxication mortelle par le harmel (Peganum harmala L.). Phytotherapie.

[11] Balíková M. (2002). Collective poisoning with hallucinogenous herbal tea. Forensic Sci. Int..

[12] O’Mahony Carey S. (2010). Psychoactive Substances: A Guide to Ethnobotanical Plants and Herbs, Synthetic Chemicals, Compounds and Products.

[13] Ott J. (1994). Ayahuasca Analogues: Pangæan Entheogens.

[14] Decree Regulating the Use of Vegetable Substances and Preparations in Food Supplements, Replacing the Decree of the Minister for Health of 9 July 2012—‘BELFRIT’—European Commission. https://ec.europa.eu/growth/tools-databases/tris/en/search/?trisaction=search.detail&year=2017&num=276.

[15] Ahmad A., Husain A., Mujeeb M., Khan S.A., Najmi A.K., Siddique N.A., Damanhouri Z.A., Anwar F. (2013). A review on therapeutic potential of Nigella sativa: A miracle herb. Asian Pac. J. Trop. Biomed..

[16] Monsef-Esfahani H.R., Faramarzi M.A., Mortezaee V., Amini M., Rouini M.R. (2008). Determination of harmine and harmaline in Peganum harmala seeds by high-performance liquid chromatography. J. Appl. Sci..

[17] Wang Z., Kang D., Jia X., Zhang H., Guo J., Liu C., Meng Q., Liu W. (2018). Analysis of alkaloids from Peganum harmala L. sequential extracts by liquid chromatography coupled to ion mobility spectrometry. J. Chromatogr. B: Anal. Technol. Biomed. Life Sci..

[18] Ministry of Health—Gazzetta Ufficiale. https://www.gazzettaufficiale.it/eli/id/2018/09/26/18A06095/SG.

[19] Dubey P.N., Singh B., Mishra B.K., Kant K., Solanki R.K. (2016). Nigella (Nigella sativa): A high value seed spice with immense medicinal potential. Indian J. Agric. Sci..

[20] Ramadan M.F. (2007). Nutritional value, functional properties and nutraceutical applications of black cumin (Nigella sativa L.): An overview. Int. J. Food Sci. Technol..

[21] Barthlott W., Ehler N. (1977). Raster-Elektronenmikroskopie der Epidermis-Oberflächen von Spermatophyten.

[22] Semerdjieva I., Yankova-Tsvetkova E. (2017). Pollen and seed morphology of Zygophylum fabago and Peganum harmala (Zygophyllaceae) from Bulgaria. Phyton Int. J. Exp. Bot..

[23] Heiss A.G., Kropf M., Sontag S., Weber A. (2011). Seed morphology of Nigella s.l. (Ranunculaceae): Identification, diagnostic traits, and their potential phylogenetic relevance. Int. J. Plant Sci..

[24] Thakur V.V., Tiwari S., Tripathi N., Tiwari G. (2019). Molecular identification of medicinal plants with amplicon length polymorphism using universal DNA barcodes of the atpF–atpH, trnL and trnH–psbA regions. 3 Biotech.

[25] Cornara L., Borghesi B., Canali C., Andrenacci M., Basso M., Federici S., Labra M. (2013). Smart drugs: Green shuttle or real drug?. Int. J. Legal Med..

[26] Ekor M. (2014). The growing use of herbal medicines: Issues relating to adverse reactions and challenges in monitoring safety. Front. Pharmacol..

[27] Health W., Geneva O. (2005). National Policy on Traditional Medicine and Regulation of Herbal Medicines Report of a WHO Global survey. https://apps.who.int/iris/handle/10665/43229.

[28] Khare B., Mishra M.K., Kesharwani L. (2018). Screening of adulterants in herbal formulations for forensic considerations. J. Pharmacogn. Phytochem..

[29] Prakash O., Kumar A., Kumar P., Kumar Manna N. (2013). Adulteration and Substitution in Indian Medicinal Plants: An Overview. J. Med. Plants Stud..

[30] Ichim M.C., Häser A., Nick P. (2020). Microscopic Authentication of Commercial Herbal Products in the Globalized Market: Potential and Limitations. Front. Pharmacol..

[31] Chieco C., Rotondi A., Morrone L., Rapparini F., Baraldi R. (2013). An ethanol-based fixation method for anatomical and micro-morphological characterization of leaves of various tree species. Biotech. Histochem..

