# Supplementary material for: Potential Risks of Plant Constituents in Dietary Supplements: Qualitative and Quantitative Analysis of Peganum harmala Seeds
Source: Molecules. 2021 Mar 4;26(5):1368. doi: 10.3390/molecules26051368 (PMC7961586; doi:10.3390/molecules26051368)
Supplement: Supplementary file 1 [file molecules-26-01368-s001.pdf]

A

First candidate

	Sequence	Lenght	Start nt	Finish nt
Forward primer	TTTTCGA GGACAAA TTCTCACA TTT	24	473	497
Reverse primer	AGGGTTT GAACCAG GATTTCG	21	540	561
Probe	TATGTGTT AGATGTA CTAATACC CT	25	502	527

```
   1 actgtatcgc actatgtgtc atttgataac tcaaaaatc ccttaccctt gttttaaatc
  61 gaattgcaaa tggaggaatt tcaagtatat ttagaactag atagatctcc gcaacgtaac
 121 ttcctatacc cacttctttt tcgggagtat atttatgcac ttgctcatga tcatggttta
 181 aatagatcga tgatttcatt ggaaaatagg ggttatgaca ataaatttag ttcactaagt
 241 gtgaaacgtt taattattcg aatgtatcaa ccgattcatt tgagtatttc tgctaattat
 301 tctaagcaaa atcaattttt tgggcacaac aataatttgt attctcaaat gatatcagag
 361 ggatttgcag tcattatgga aattccattt tccctaagat tcgtatcttt cttagaaagg
 421 aaagaaatag caaaatctca aaatttccaa tcaattcatt caatatttcc ttttttcgag
 481 gacaaattct cacatttaaa ttatgtgtta gatgtactaa taccctaccc catttgcccc
 541 gaaatcctgg ttcaaaccct tcgctactgg gtaaaagacg cctcctcttt acatttatta
 601 cggttctttc tccatgagta ttttaattgg aatggtctta ttactccaaa aaaatctatt
 661 actatttttt caaaaagtaa tccaagatta ttcttgttcc tatataattc tcatctctgt
 721 gaatatgaat ctatcttctt ttttatccgt aaccaatcgt ctcatttacg atcaacatct
 781 tctggagtct ttcttgagcg aacatattta tatggaaaag tagaatatct tggcgaagcc
 841 gttgctaatg attttcagga catcttatgt ttgttcaagg atcctttcct gcattatgtt
 901 agatatcaag gaaaatctat tctggcttca aaagatacgc ctcttctgat gaataaatgg
 961 aaatattact ttgtcaattt atggcaatgg cattttcatg tgtggtcgca accaggaagg
1021 gtccatataa atcacctatc caaacattct atcaactttc tgggctatct ttccaatgtg
1081 caactaaatc cttgggtggt acggactcaa atgttagaaa attcatttct aatagataat
1141 gttatgaaga agttcgatac aaacgttccc gttattcctc tgattggatc attgactaag
1201 gcggggtttt gtaacacatt agggcatccc attagtaagc cgacctgggc cgattcctcc
1261 gattctcata ttatcgagcg atttgtgcgt atatgcagaa atctttctca ttatcacagt
1321 ggatcctcaa aaaaaaagag tttgtatcga ataaaatata tccttcggct ttcttgtgtt
1381 aaaagtttgg ttcgtaaaca caaaagtact gtacgcgtct ttttgaaaag attaggttcg
1441 gaattctcgg aagaattcct tacggaggaa gagcacgttc tttctttaat ctttccagga
1501 gcttcgttta cttcgcggag gttatataga gggcggattt ggtatttgga tattatttgt
1561 ataaatgatc tggttaatca ggatcatgaa tgattggtta tgagaccgcg taagttttat
1621 aaatttcttt aaatggtgaa tagataaaaa aaattcattt atttcgatta aatgttcatg
1681 caataaaaat aataaaggtt gatgaactga gtattccgct tttttagtgt atttttaggg
1741 aagaaacaga gttttcgatg ttatacatag ggaaagccgt gtgcaatgaa aaatgcaagc
1801 acgg
```

B

Second candidate

	Sequence	Lenght	Start nt	Finish nt
Forward primer	TTTTCGA GGACAAA TTCTCACA TTT	24	473	497
Reverse primer	CGAAGGG TTTGAAC CAGGATT	21	543	564
Probe	TATGTGTT AGATGTA CTAATACC CT	25	509	534


```
   1 actgtatcgc actatgtgtc atttgataac tcaaaaatc ccttaccctt gttttaaatc
  61 gaattgcaaa tggaggaatt tcaagtatat ttagaactag atagatctcc gcaacgtaac
 121 ttcctatacc cacttctttt tcgggagtat atttatgcac ttgctcatga tcatggttta
 181 aatagatcga tgatttcatt ggaaaatagg ggttatgaca ataaatttag ttcactaagt
 241 gtgaaacgtt taattattcg aatgtatcaa ccgattcatt tgagtatttc tgctaattat
 301 tctaagcaaa atcaattttt tgggcacaac aataatttgt attctcaaat gatatcagag
 361 ggatttgcag tcattatgga aattccattt tccctaagat tcgtatcttt cttagaaagg
 421 aaagaaatag caaaatctca aaatttccaa tcaattcatt caatatttcc ttttttcgag
 481 gacaaattct cacatttaaa ttatgtgtta gatgtactaa taccctaccc catttgcccc
 541 gaaatcctgg ttcaaaccct tcgctactgg gtaaaagacg cctcctcttt acatttatta
 601 cggttctttc tccatgagta ttttaattgg aatggtctta ttactccaaa aaaatctatt
 661 actatttttt caaaaagtaa tccaagatta ttcttgttcc tatataattc tcatctctgt
 721 gaatatgaat ctatcttctt ttttatccgt aaccaatcgt ctcatttacg atcaacatct
 781 tctggagtct ttcttgagcg aacatattta tatggaaaag tagaatatct tggcgaagcc
 841 gttgctaatg attttcagga catcttatgt ttgttcaagg atcctttcct gcattatgtt
 901 agatatcaag gaaaatctat tctggcttca aaagatacgc ctcttctgat gaataaatgg
 961 aaatattact ttgtcaattt atggcaatgg cattttcatg tgtggtcgca accaggaagg
1021 gtccatataa atcacctatc caaacattct atcaactttc tgggctatct ttccaatgtg
1081 caactaaatc cttgggtggt acggactcaa atgttagaaa attcatttct aatagataat
1141 gttatgaaga agttcgatac aaacgttccc gttattcctc tgattggatc attgactaag
1201 gcggggtttt gtaacacatt agggcatccc attagtaagc cgacctgggc cgattcctcc
1261 gattctcata ttatcgagcg atttgtgcgt atatgcagaa atctttctca ttatcacagt
1321 ggatcctcaa aaaaaaagag tttgtatcga ataaaatata tccttcggct ttcttgtgtt
1381 aaaagtttgg ttcgtaaaca caaaagtact gtacgcgtct ttttgaaaag attaggttcg
1441 gaattctcgg aagaattcct tacggaggaa gagcacgttc tttctttaat ctttccagga
1501 gcttcgttta cttcgcggag gttatataga gggcggattt ggtatttgga tattatttgt
1561 ataaatgatc tggttaatca ggatcatgaa tgattggtta tgagaccgcg taagttttat
1621 aaatttcttt aaatggtgaa tagataaaaa aaattcattt atttcgatta aatgttcatg
1681 caataaaaat aataaaggtt gatgaactga gtattccgct tttttagtgt atttttaggg
1741 aagaaacaga gttttcgatg ttatacatag ggaaagccgt gtgcaatgaa aaatgcaagc
1801 acgg
```


C

Third candidate

	Sequence	Lenght	Start nt	Finish nt
Forward primer	TGTGGTC GCAACCA GGAA	18	1010	1018
Reverse primer	CCCAAGG ATTTAGTT GCACATT G	23	1074	1097
Probe	TCCATATA AATCACCT ATCCAAA CA	25	1022	1047


```
   1 actgtatcgc actatgtgtc atttgataac tcaaaaatc ccttaccctt gttttaaatc
  61 gaattgcaaa tggaggaatt tcaagtatat ttagaactag atagatctcc gcaacgtaac
 121 ttcctatacc cacttctttt tcgggagtat atttatgcac ttgctcatga tcatggttta
 181 aatagatcga tgatttcatt ggaaaatagg ggttatgaca ataaatttag ttcactaagt
 241 gtgaaacgtt taattattcg aatgtatcaa ccgattcatt tgagtatttc tgctaattat
 301 tctaagcaaa atcaattttt tgggcacaac aataatttgt attctcaaat gatatcagag
 361 ggatttgcag tcattatgga aattccattt tccctaagat tcgtatcttt cttagaaagg
 421 aaagaaatag caaaatctca aaatttccaa tcaattcatt caatatttcc ttttttcgag
 481 gacaaattct cacatttaaa ttatgtgtta gatgtactaa taccctaccc catttgcccc
 541 gaaatcctgg ttcaaaccct tcgctactgg gtaaaagacg cctcctcttt acatttatta
 601 cggttctttc tccatgagta ttttaattgg aatggtctta ttactccaaa aaaatctatt
 661 actatttttt caaaaagtaa tccaagatta ttcttgttcc tatataattc tcatctctgt
 721 gaatatgaat ctatcttctt ttttatccgt aaccaatcgt ctcatttacg atcaacatct
 781 tctggagtct ttcttgagcg aacatattta tatggaaaag tagaatatct tggcgaagcc
 841 gttgctaatg attttcagga catcttatgt ttgttcaagg atcctttcct gcattatgtt
 901 agatatcaag gaaaatctat tctggcttca aaagatacgc ctcttctgat gaataaatgg
 961 aaatattact ttgtcaattt atggcaatgg cattttcatg tgtggtcgca accaggaagg
1021 gtccatataa atcacctatc caaacattct atcaactttc tgggctatct ttccaatgtg
1081 caactaaatc cttgggtggt acggactcaa atgttagaaa attcatttct aatagataat
1141 gttatgaaga agttcgatac aaacgttccc gttattcctc tgattggatc attgactaag
1201 gcggggtttt gtaacacatt agggcatccc attagtaagc cgacctgggc cgattcctcc
1261 gattctcata ttatcgagcg atttgtgcgt atatgcagaa atctttctca ttatcacagt
1321 ggatcctcaa aaaaaaagag tttgtatcga ataaaatata tccttcggct ttcttgtgtt
1381 aaaagtttgg ttcgtaaaca caaaagtact gtacgcgtct ttttgaaaag attaggttcg
1441 gaattctcgg aagaattcct tacggaggaa gagcacgttc tttctttaat ctttccagga
1501 gcttcgttta cttcgcggag gttatataga gggcggattt ggtatttgga tattatttgt
1561 ataaatgatc tggttaatca ggatcatgaa tgattggtta tgagaccgcg taagttttat
1621 aaatttcttt aaatggtgaa tagataaaaa aaattcattt atttcgatta aatgttcatg
1681 caataaaaat aataaaggtt gatgaactga gtattccgct tttttagtgt atttttaggg
1741 aagaaacaga gttttcgatg ttatacatag ggaaagccgt gtgcaatgaa aaatgcaagc
1801 acgg
```

Identification of species-specific *matk* region

Figure S1. Three candidates amplicons for *Pgh matk* detection. The forward and reverse primers are coloured in blue, while the FAM-TAMRA probe is marked in red. The chosen amplicon is described in figure S1 C.
